# Protein Retention Assessment of Four Levels of Poultry By-Product Substitution of Fishmeal in Rainbow Trout (*Oncorhynchus mykiss*) Diets Using Stable Isotopes of Nitrogen (δ^15^N) as Natural Tracers

**DOI:** 10.1371/journal.pone.0107523

**Published:** 2014-09-16

**Authors:** Daniel Badillo, Sharon Z. Herzka, Maria Teresa Viana

**Affiliations:** 1 Programa de Maestría y Doctorado en Ecología Molecular y Biotecnología, Facultad de Ciencias Marinas, Universidad Autónoma de Baja California (UABC), Ensenada, B.C., México; 2 Departamento de Oceanografía Biológica, Centro de Investigación Científica y de Educación Superior de Ensenada, Carretera Ensenada, Tijuana No., Ensenada, B.C., México; 3 Instituto de Investigaciones Oceanológicas, UABC, Ensenada, B.C., México; Center for Nanosciences and Nanotechnology, Mexico

## Abstract

This is second part from an experiment where the nitrogen retention of poultry by-product meal (PBM) compared to fishmeal (FM) was evaluated using traditional indices. Here a quantitative method using stable isotope ratios of nitrogen (δ^15^N values) as natural tracers of nitrogen incorporation into fish biomass is assessed. Juvenile rainbow trout (*Oncorhynchus mykiss*) were fed for 80 days on isotopically distinct diets in which 0, 33, 66 and 100% of FM as main protein source was replaced by PBM. The diets were isonitrogenous, isolipidic and similar in gross energy content. Fish in all treatments reached isotopic equilibrium by the end of the experiment. Two-source isotope mixing models that incorporated the isotopic composition of FM and PBM as well as that of formulated feeds, empirically derived trophic discrimination factors and the isotopic composition of fish that had reached isotopic equilibrium to the diets were used to obtain a quantitative estimate of the retention of each source of nitrogen. Fish fed the diets with 33 and 66% replacement of FM by PBM retained poultry by-product meal roughly in proportion to its level of inclusion in the diets, whereas no differences were detected in the protein efficiency ratio. Coupled with the similar biomass gain of fishes fed the different diets, our results support the inclusion of PBM as replacement for fishmeal in aquaculture feeds. A re-feeding experiment in which all fish were fed a diet of 100% FM for 28 days indicated isotopic turnover occurred very fast, providing further support for the potential of isotopic ratios as tracers of the retention of specific protein sources into fish tissues. Stable isotope analysis is a useful tool for studies that seek to obtain quantitative estimates of the retention of different protein sources.

## Introduction

Feed production for aquaculture is highly dependent on protein ingredient supplies, especially on fishmeal, which is considered the primary source of protein. According to Tacon and Metian [1], 75% of the world's fish stocks are currently considered as fully exploited or overexploited, including many small pelagic fish species used to produce fishmeal for feed formulation worldwide. Since fishmeal production is forecast to be unable to support the growth of the aquaculture sector, the search for alternative ingredients and protein sources and the optimization of dietary protein content is an important goal [2].

Dietary protein has numerous structural and metabolic functions that are essential for sustaining fish growth, structural body composition, muscle contraction, cell signalling and for ensuring the adequate function of the cell cycle [3]. Since particular metabolic functions require specific amino acids, it is crucial that fish ingest, digest and assimilate the necessary amino acids from protein sources. Protein quality is therefore generally evaluated according to its amino acid content. Its quality is often tested based on the assessment of digestive capacity using *in vitro* digestibility assays performed with a pH Stat on crude homogenates, using digestive enzymes extracted from the species of interest [4], [5]. Although these *in vitro* experiments provide information on digestive capability of particular dietary components, they cannot be used to relate protein retention with growth.

The traditional methodologies employed in the study of protein utilization, including feed ingestion, growth, and *in vivo* apparent digestibility rely on *in vivo* experiments under controlled laboratory conditions. Complicated physiological laboratory experiments are necessary to measure energy expenditure and ammonia production to estimate the amount of protein used to support metabolic functions and as an energy source [6]. Results from *in vitro* experiments have been criticized for not being reproducible under commercial conditions [2]. Moreover, laboratory experiments are usually performed only for short periods of time and usually focus on earlier life stages than those used in commercial production [2].

Digestibility depends on the availability of specific digestive enzymes that break down the protein into small peptides. The availability of specific proteins (related to their degree of solubility) is crucial, as is the availability of certain amino acids for enzymatic cleavage in order to produce specific peptides for further digestion into free amino acids that are absorbable within few hours. Thus, diets with similar crude protein content that differ in protein sources or that contain proteins that have undergone various types of processing can result in different retention performance. Methodologies that can provide quantitative estimates of the differential retention of alternative protein sources used in diet formulations under purely experimental and commercial conditions are thus a valuable tool.

Poultry by-product meal (PBM) is an ideal protein source for the partial substitution of fishmeal. According to Tacon and Metian [1], PBM substitutes up to 30% of fishmeal in diets for salmon, sea trout and shrimp. Australia, Canada, Chile and Mexico are the main users of PBM. Partial replacement of fishmeal with PBM has been shown to yield a similar growth performance than that obtained solely with fishmeal in aquaculture diets [2], [7]. For example, an 80% replacement of fishmeal by PBM in shrimp diets and a 30% replacement in rainbow trout diets yielded a similar growth performance than in those fed a diet with fishmeal as the only protein source [8], [9].

Measurements of the relative abundance of the stable isotope of carbon (^13^C/^12^C, expressed as δ^13^C values) and nitrogen (^15^N/^14^N, δ^15^N values) have been used for dietary reconstruction in ecological studies because the isotopic composition of tissues reflects that of an animal's diet [10], [11]. Stable isotope analysis has also been used to distinguish between protein sources and to estimate their relative contribution to an animal's biomass and to infer food quality [12], [13], [14] or determining the length of time an individual has spent in a new environment [15]. The contribution of different protein sources into diets to biomass production can be estimated using isotope-mixing models [16], [17], [18]. However, few studies have used stable isotopes to quantify the retention of alternative protein sources to fishmeal in fish nutrition.

Estimating the contribution of different protein sources included in diets to an animal's biomass using isotope-mixing models has three requirements. First, the protein sources must have distinct isotopic compositions [19]. Second, the isotopic composition of consumer tissues must be corrected for trophic discrimination. There is enrichment in the heavy isotope relative to a consumer's diet due amino acid deamination and transamination [20]. Ideally, trophic discrimination factors need to be estimated empirically on a tissue and species-specific basis under controlled conditions and incorporated into mixing models [21], [22], [23]. If protein quality varies among diets, then trophic discriminations factors need to be estimated for each formulation [24]. Third, an individual's tissues must be in isotopic equilibrium with the diet. Following a shift to a diet of different isotopic composition, the isotopic composition of soft tissues will gradually reflect that of the new diet [25], [26]. Experimentally, this implies that animals must be fed a diet of constant isotopic composition until the tissue of interest fully reflects that of the diet. For teleost fishes, a six to eight-fold increase in biomass while fed a single diet of constant isotopic composition can be considered sufficient to ensure that isotopic equilibrium has been closely approximated [27].

In a previous experiment, we demonstrated that the partial replacement of fishmeal with PBM was achievable without a detrimental effect on growth [28]. However, the level of retention of each protein source as a function of the fishmeal replacement level was not quantified. Thus, the aim of this study was to quantitatively estimate the nitrogen retention of diets in which poultry by-product meal was used to replace fishmeal at four different levels. We used juvenile rainbow trout (*Oncorhynchus mykiss*) as a model species and the stable isotopes of nitrogen as natural tracers of diet retention. We capitalized on differences in the nitrogen isotope ratios of diets with different formulations and applied a two source isotope-mixing model to estimate the percentage of incorporation of these two protein sources into muscle tissue following an 80 d feeding experiment. In addition, we evaluated whether fish fed a finishing diet of fishmeal reflected its isotopic composition following a 28 d re-feeding period.

## Methods

### Dietary treatments

Four isonitrogenous (43.5% crude protein) and isolipidic (12.5% crude fat) experimental diets with a gross energy ranging from 19.3 to 21.2 kJ g^−1^ were formulated with graded levels of poultry by-product meal (PBM). Dietary treatments included 0, 33, 67 and 100% PBM (Table 1). Commercial gelatin (80% protein content) was used as binder (6% by weight in all diets) with corresponds to a contribution of 10% of the N. Fish oil (FO) and poultry oil (PO) were use to maintain a proportional ingredient substitution. The USA National Renderers Association supplied “pet food grade” PBM and PO. The fishmeal (60.0% crude protein made from tuna fish by-products) was acquired from Proteínas Marinas y Agropecuarias SA de CV (Guadalajara, Mexico). Starch and gelatine were cooked separately and added to the ingredients and blended to produce a homogeneous mixture with a 60% moisture content. The resulting mixture in the form of dough was then cold-pressed through a meat grinder into 0.2 mm diameter strips, from which pellets were cut and then dried at 60°C for 24 h. The feed was then stored at −30°C until it was fed to the fish.

**Table 1:** Ingredients (g per Kg), proximate composition on a dry weight basis (%) and δ^15^N values of four experimental diets containing different levels of substitution of fishmeal with poultry by-products meal (PBM; 0, 33, 67 and 100% replacement).

	TREATMENTS			
Ingredients	0PBM	33PBM	67PBM	100PBM
Poultry by-product meal (PBM)^1^	0.0 (0.0)	235 (0.3)	440 (0.6)	590 (0.9)
Fishmeal^2^	660 (0.9)	400 (0.5)	175 (0.2)	0 (0.0)
Corn meal	55 (0.01)	55 (0.01)	55 (0.01)	55 (0.01)
Poultry oil^3^	0.0	5.0	17	35
Fish oil^4^	72	52	28	0
Corn starch	91	131	166	201
Gelatin	60 (0.1)	60 (0.1)	60 (0.1)	60 (0.1)
Rovimix for carnivores fish^5^	30	30	30	30
Stay c^5^	4	4	4	4
Sodium Benzoate	2.3	2.3	2.3	2.3
Choline chloride	0.9	0.9	0.9	0.9
Tocopherol	0.1	0.1	0.1	0.1
Cellulose	25	25	25	25
TOTAL	1000	1000	1000	1000
***Proximate composition (% of dry matter)***				
Crude protein (%)	43.1	43.4	43.7	43.6
Crude fat (%)	12.5	12.5	12.5	12.3
Ash (%)	19.8	15.1	12.1	8.8
NFE^6^	24.5	29.0	31.7	35.3
***Diet isotopic composition***				
δ^15^N (‰)	12.7	9.0	6.1	4.2

The four diets were fed to juvenile rainbow trout (*Oncorhynchus mykiss*) for 80 d. Fractional N contribution is expressed inside parentheses ( ).

1 “Pet food grade” (69.6% crude protein, 12% crude lipid) supplied by the National Renderers Association, USA.

2 Tuna fishmeal (60.0% crude protein, 6.9% crude lipid) from Proteínas Marinas y Agropecuarias, Guadalajara, México.

3 Poultry oil from Proteínas Marinas y Agropecuarias, Guadalajara, México.

4 Sardine oil.

5 Rovimix; Stay–C donated by DSM, Guadalajara, México.

6 Nitrogen free extract: calculated by difference: NFE  =  100-CP-CF-Ash.

δ^15^N values of main nitrogen-containing ingredients: Fishmeal (14.1‰), poultry by-product meal (3.4‰) and gelatin (7.8‰).

### Experimental design

Juvenile rainbow trout (*Oncorhynchus mykiss*) used during feeding experiments were held the Fish Nutrition Experimental Laboratory at the Universidad Autónoma de Baja California (UABC). Experiments were performed according to the ethics guidelines of the university and in accordance to international guidelines, and this study was approved and supervised by the ethics committee of the Instituto de Investigaciones Oceanológicas (IIO, UABC). The fertilized ova were obtained from Troutlodge Inc. (Orting, WA) and transported by plane to the research facility in Mexico.

Prior to the feeding experiments, all fish were fed the control (0PBM) diet formulated with fishmeal as the only protein source for one month. After the one-month period, 600 fish (1.4±0.1 g) were randomly distributed in 12 experimental units consisting of 500L fiberglass tanks with 50 fish each. The four dietary treatments were evaluated in triplicate. Tanks were connected to a biofilter coupled to a recirculation system and set to 5% water renewal every day. The biofilter consisted of a 200L Pneumatic Drop Bead Filter, a plastic compensation tank (1,100L) and a pump (0.7 HP). Water was maintained at 14.0±1°C by cooling the water in a reservoir after filtration through the biofilter. Water was monitored twice a week to ensure adequate levels of ammonia, nitrites, nitrates, pH and carbonate using a kit master for fresh water.

Fish were fed the experimental diets to apparent satiation 7 days a week at 0800, 1200, 1600 and 2000 hrs. The fish in each tank were weighted and counted every 20 days and the quantity of feed consumed was registered daily. Tanks were cleaned and siphoned daily and survival was continuously monitored. After 80 d, three fish from each tank were euthanized by hypothermia in accordance with the University's policy on health and safety and based on the approval from the ethics supervising committee. Muscle samples from the dorsal to the caudal fin region of each of three fish from each experimental unit (tank) were dissected, pooled and stored at -80°C pending isotopic analysis. Hence, each replicate consists of a pooled sample of three fish from a single tank.

A second experiment was conducted following completion of the 80 d feeding trial to evaluate how quickly fish incorporated fishmeal into their muscle (finalization diet). All groups were re-fed with the diet 0% PBM for 4 more weeks. On days 0, 14, 21 and 28, three fish from each tank were randomly selected and euthanized and muscle tissue was dissected (n = 3 pooled samples per treatment) and prepared for stable isotope analysis as described above.

The following indices were calculated to evaluate growth performance:
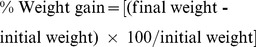
(1)


Thermal growth coefficient (TGC)
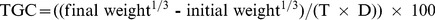
(2)


### Protein efficiency ratio



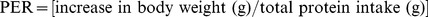
(3)


### Proximate analysis

The proximate composition of each diet was measured (in triplicate) and expressed on a dry matter basis according to standard procedures [29]. The moisture content of each sample was calculated from samples (2g) and dried to constant weight at 60°C. Total nitrogen content was determined with the micro-Kjeldahl method, and percent crude protein was then calculated as% N x 6.25. Total lipid concentration was determined by Soxhlet extraction with petroleum ether as a solvent and the crude fat was calculated gravimetrically. Ash content was determined by heating samples to 550°C for 6 h. The nitrogen-free extract was calculated by difference (% NFE  =  100 − (% crude protein +% total lipid +% ash).

### Isotopic analysis

The δ^15^N values of dry, defatted feed samples, fishmeal, poultry by product-meal and muscle tissue were determined. Samples were dried (60°C for 24 h) and homogenized with a mortar en pestle. About 1.5±0.5 mg were weighed using an ultrabalance (±0.1 µg), placed into tin capsules and sent to the Stable Isotope Facility of the University of Davies California (USA) for isotopic analysis. The laboratory's internal standards had a standard deviation of ≤ 0.3‰ for ^15^N. Isotope values are expressed in delta (δ) notation in parts per thousand (‰) relative to atmospheric N_2_ as follows:
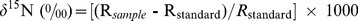
(4)where, R sample and R standard are the ratio of heavy to light isotopes (^15^N/^14^N) in the sample and standard, respectively. Trophic discrimination factor of muscle tissue (**Δ**) relative to each experimental diet at isotopic equilibrium was calculated as follows: 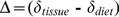
(5)


A two-source isotope-mixing model was used to estimate the fraction of each source that was differentially retained into muscle tissue at isotopic equilibrium [30]:
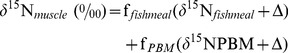
(6)




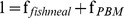
(7)


δ^15^N_muscle_, δ^15^N_fishmeal_ and δ^15^N_PBM_ are the isotopic composition of fish muscle, fishmeal and poultry by-product meal, respectively, and f_fishmeal_ and f_PBM_ are the fraction of nitrogen retained from the fishmeal and poultry by product meal. δ^15^N_muscle_ values were corrected for trophic discrimination using the mean value calculated for muscle tissue equilibrated onto the 33 and 67PBM diets at the end of the 80 d feeding period. To evaluate the potential impact of natural variation in trophic discrimination factors on the results of the mixing model, we performed a sensitivity analysis. The mixing models were also run using the trophic discrimination factors calculated for fish at isotopic equilibrium with the 0PBM and 100 PBM diets and. For the 0% and 100% PBM, the isotopic composition of the trout muscle at isotopic equilibrium reflects the diet that includes only fishmeal or poultry by-product meal, respectively, and the use of mixing models is unnecessary.

### Statistical analysis

Differences among treatments in biological indices and trophic discrimination values calculated for the muscle tissue from each experimental unit were evaluated using a one-way analysis of variance (ANOVA; n = 3). When statistical differences were detected a Tukey post-hoc test was used to identify statistical differences between treatments. SigmaStat for Windows 3.5 was used to perform the statistical analysis.

## Results

After being fed for 80 d on the four experimental diets, no significant differences were detected (P<0.05) in TGC based on the results of the one-way ANOVA; values were around 1.5 (Table 2). The PER was also similar among treatments with values around 2.8 g body weight g protein intake^−1^. However, fish fed the 67% PBM diet had a significantly higher final weight than the rest of the treatments, whereas the 100% PBM had a significantly lower final weight than the other treatments. Nevertheless, the total percent weight gain relative to initial weight ranged from 1480 to 1587%, indicating that isotopic equilibrium was reached in all treatments.

**Table 2:** Biological indices, nitrogen isotope ratios (δ^15^N) of muscle tissue and trophic discrimination values for juvenile rainbow trout (*Oncorhynchus mykiss*) fed four experimental diets formulated to contain similar protein and lipid levels.

	Experimental Treatments			
	0PBM	33PBM	67PBM	100PBM
***Biological indices***				
Final weight (g)	21.8±1.5^ab^	21.9±1.1^ab^	23.5±1.6^a^	20.9±0.5^b^
TGC^1^	1.48±0.05	1.48±0.01	1.54±0.05	1.47±0.02
Weight gain (% of initial)	1480±39	1495±32	1587±32	1495±103
PER^2^	2.85±0.1	2.72±0.3	2.78±0.2	2.77±0.1
***Isotope ratio of muscle at equilibrium with the diet***				
δ^15^N (‰)	14.7±0.9^a^	10.1±0.1^b^	7.1±0.1^c^	4.1±0.0^d^
***Isotopic trophic discrimination***				
Δδ^15^N (‰)	2.0±0.1^a^	1.1±0.1^b^	1.0±0.1^b^	−0.1±0.0^d^

Fishmeal and fish oil were incrementally substituted for poultry by-product meal (PBM; 0, 33, 67 and 100%) and poultry oil. Measurements were made after 80 d. Values within the same row with different superscripts letters were significantly different (P<0.05).^1^ 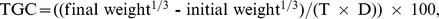 [43] ^2^ 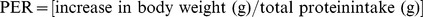.

After 80 days, the muscle tissue of the fish had isotopic values similar to that of each diet. The trophic discrimination factors differed significantly between treatments. For nitrogen, Δδ^15^N ranged between 2.0‰ for fish fed the 0%PBM and −0.1‰ for those fed the 100%PBM, while Δδ^15^N was similar for the 33PBM and 67PB treatments, whereas the fish from the 67% PBM treatment assimilated 78% of their nitrogen from PBM (Table 3). Using meal-based endpoints yielded a 5% larger and a 3% smaller retention of PBM for fish fed the 33% and 67%PBM diets, respectively.

**Table 3:** Percentage of nitrogen retention by rainbow trout (*Oncorhynchus mykiss*) fed formulated diets with two levels (33 and 67%) replacement of fishmeal (FM) with poultry by-product meal (PBM).

	Treatments	
	33PBM	67PBM
	TD = 1.1‰ (−0.1‰; 2.0‰)	TD = 1.1‰; (−0.1‰; 2.0‰)
(a) Diet-based endpoints		
Fishmeal	64.1 (77.6; 52.9)	21.8 (35.3; 10.6)
PBM	35.9 (22.4; 47.1)	78.2 (64.7; 89.4)
b) Meal-based endpoints considering two ingredients		
Fishmeal	58.4 (30.8; 49.5)	24.8 (35.3; 15.9)
PBM	41.6 (69.2; 50.5)	75.5 (64.5; 84.1)

Nitrogen retention was estimated using a simple two-source mixing model considering (a) the isotopic composition of the diets formulated with only fishmeal or poultry by-product meal as endpoints and (b) the isotopic composition of the fishmeal and poultry by-product meal itself. The mixing model was applied after correcting for trophic discrimination (TD) using the average value calculated for fish equilibrated onto the 33 and 67PBM diets (1.1 ‰). Results of a sensitivity analysis of mixing model output to variations in TD are reported in parentheses.

The results of the sensitivity analysis indicated that in most cases, variations ca. ± 1 ‰ in Δδ^15^N led to differences in the percent of nitrogen retention that ranged 8.9 and 13.5% (Table 3). The one exception was for the 33PBM, using the meal-based endpoints and a lower estimate of TD. In that case, the contribution of PBM was 28% higher.

**Figure 1:**
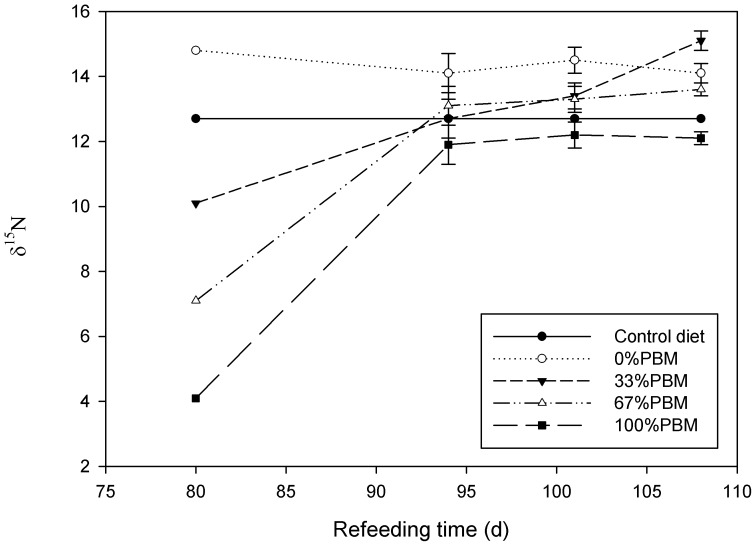
Isotopic values (δ^15^N; ‰) of rainbow trout (*Oncorhynchus mykiss*) muscle tissue that were isotopically equilibrated into four experimental diets with different levels of substitution of fishmeal with poultry by-product meal (time 0) and were then switched to the control diet and fed for a 28 additional days. PBM: Poultry by product meal. Values shown are means (n = 3) ±SD.

After the fish from the four experimental diets were switched to the diet containing only fishmeal for 28 days, the isotopic composition of muscle tissue changed quickly toward the value of the 0PBM diet (Figure 1). Fish from the 33PBM, and 67PBM had isotopic values of 15.1 and 13.6 ‰ after 28 days, whereas the treatment 100PBM reached a value of 12.0 ‰ after 21 days.

## Discussion

Stable isotopes have proven very useful as natural tracers for calculating the relative contribution of multiple food or carbon sources in an animal's diet to its biomass [10], [31], [32]. In spite of increasing number of nutrition experiments in which stable isotopes are used, only few of them are focused on the fishmeal substitution as protein source. [33] The potential applications of this technique to nutritional studies are ample and can provide quantitative estimates of the retention of different protein sources into a consumer's tissues that are difficult to obtain with other techniques, especially in the case of carnivorous fish that require large amounts of protein in formulated diets.

In nutritional experiments it is widely recommended that a 400% increase in weight should be achieved to assess the effect of an ingredient or additive on an organism's performance [2]. In the case of the isotopic composition of an organism's tissues, the time needed to achieve an isotopic equilibrium, a pre-requisite to the application of mixing models, depends on the rate of isotopic turnover. For muscle tissue of teleosts, the rate of isotopic turnover is related to growth rate, particularly in young and rapidly growing organisms [26], [34], [35], [17]. At the beginning of the experiment, juvenile rainbow trout weighted 1.4±0.1 g wet weight. After 80 d the fish had increased in weight about 15-fold, indicating that full isotopic equilibrium with the diets was reached [27]. This ensured that the application of the isotope-mixing model could be used to calculate the fraction of PBM incorporated into the trout's muscle tissue.

The nitrogen isotope discrimination factors calculated for fish fed the four formulated diets ranged between 2 and -0.1‰, despite the fact that the diets were isonitrogenous, isolipidic and similar in gross energy content. The highest trophic discrimination factor was found for fish in isotopic equilibrium with the 100% fishmeal diet, while there was little or no discrimination observed for fish fed the 100% PBM diet. Pares et al. [28] analysed the effect of fatty acids and amino acid composition of the diets used in this study, and reported that their amino acid profiles were somewhat similar. Hence, the formulated diets yielded different levels of isotopic discrimination despite having similar protein content and amino acid profiles content.

All diets contained commercial gelatin as binder, a protein that was constant in all treatments with a contribution of 10% of the nitrogen contained in diets (Table 1). Gelatin was used as binder due to their capacity to bind fish diets under laboratory conditions without the addition of high quantities of carbohydrates, which are not easily tolerated by fish. Gelatin is easily assimilated by most organisms [28], [36]. Its nitrogen isotopic composition was 7.8‰, compared to 14.1‰ and 3.4‰ for FM and PBM, respectively (Table 1), which explains the difference in δ^15^N values of the meals vs. whole diets. Since PBM and FM contributed about 90% of the nitrogen in the formulated diets, differences in the results of the mixing model are assumed to be attributed mostly to the differential retention of FM and PBM in each of the two diets (i.e., the retention of gelatin is assumed to be constant). Unfortunately, we were unable to successfully estimate the fractional contribution of gelatin by incorporating δ^13^C values of the three protein sources into mixing models due to limited differences in their highly variable exploratory results with different carbon isotopic composition.

Therefore, it is suggested that PBM in trout is not only as highly digestible than FM or at least as digestible than the kind used, but is highly retained. The protein efficiency ratio (PER) calculated here fail to show differences, a calculation reported as growth increase from protein ingested, being unable to quantitatively estimate how much and from which source was retained. Even if PER measurement is considered as a sensitive indicator, here it is demonstrated that the use stable isotopes is a more sensible and accurate index for protein substitution evaluations.

Previous studies have related protein quality to the level of trophic discrimination in a consumer's tissues. Robbins et al. [24] found that the plasma of rats fed high protein diets (fishmeal) showed lower trophic discrimination values compared with those fed various plant-based diets (3.3 ‰ vs 5.0 ‰, respectively). Gaye-Seissenger et al. [13] fed Nile tilapia (*Oreochromis niloticus*) diets with a range of protein levels and did not find significant differences in nitrogen discrimination values between treatments. However, they did find that trophic discrimination values calculated for individual whole fish were negatively and linearly correlated with protein accretion. They concluded that individual protein balance could influence the level of nitrogen isotope discrimination between a consumer and its diet. More generally, Martínez del Río and Wolf [19] applied mass balance models and predicted that nitrogen isotope discrimination should be lower in animals fed diets with higher protein quality and with higher protein accretion (termed nitrogen deposition in their study). Based on a subsequent review of the literature, Martínez del Río et al. [17] concluded that the evidence accrued to date regarding the relationship between nitrogen isotope discrimination and protein content had yielded varying results, and highlighted the need for laboratory experiments. In this study, the diets were formulated to maintain a constant protein content of 43–44%, and the variation in nitrogen isotope discrimination cannot be attributed to differences in protein content.

Isotopic routing, or the channelling of specific components (in this case amino acids) assimilated from a diet into specific tissues can also influence discrimination factors, but this process is not well understood [19], [21]. The underlying mechanisms leading to differences in isotope discrimination values are therefore unclear, and identifying the causes underlying the variation in nitrogen isotope discrimination is beyond the scope of this study. Our purpose was to calculated trophic discrimination values so as to apply the isotope-mixing models and estimate the incorporation of FM vs. PBM in diets with different formulations in order to eliminate or reduce the inclusion of FM in aquafeeds. Using empirically derived trophic discrimination factors ensures the estimates of the fractional contribution of different protein sources to trout muscle tissue are robust.

According to the results of the isotope mixing model, the 33% and 67% PBM diets showed a slightly higher level of retention of nitrogen from the poultry by product meal than what was included in each formulation. The 33% and 67% PBM dietary treatments showed a 35.9 and 78.2% retention. However, the results of the sensitivity analysis indicates that variations in the value of Δδ^15^N yields a range of nitrogen retention estimates that overlaps with the level of PBM-based protein included in the diets. Although the mixing model results clearly indicate that the contribution of PBM N to fish biomass was similar to that of its level of inclusion, the uncertainly associated with Δδ^15^N makes it impossible to infer preferential retention of either fishmeal or PBM.

The apparent digestibility coefficient (ADC) of crude protein in PBM ranges between 69 and 96%, which is comparable to the ADCs of crude protein reported for various types of fishmeal [37]. A possible explanation for these results could be that differences in the overall digestibility (of protein and lipids) between protein sources or a limitation in some other essential nutrient led to a protein sparing effect.

Earlier reports have attempted to use PBM to replace fishmeal in several species, including *Micropterus salmoides* [38], *Morone chrysops X M. saxatilis* [39], [40], *Rachycentron canadum* [41] and *Trachinotus carolinus L.* [7]. Those studies reported good results in terms of growth rate. However, most did not examine the effect of the total replacement of FM by PBM. Rainbow trout fed the 100PBM treatment seems to result in similar PER.

During the 28d re-feeding experiment, the isotopic composition of the juvenile rainbow trout muscle changed quickly, indicating a very fast rate of isotopic turnover. Rapid isotopic turnover is characteristic of fishes with fast growth rate [42], which was the case of the juvenile rainbow trout used in this study. The δ^15^N values of the fish fed the 67PBM and 100 PBM were close to isotopic equilibrium by day 15 of the re-feeding period, as indicated by the consistency in the isotopic ratios of the muscle tissue on day 15 and thereafter. In contrast, the fish from the 33PBM treatment became increasingly enriched in ^15^N until the end of the experiment, suggesting that isotopic equilibrium may not have been reached in that treatment. The comparatively high level of enrichment (ca. 3 ‰) relative to the diet on day 28 after the re-feeding with the 100% FM diet may be due to isotopic routing of specific amino acids, although our data do not allow for that sort of analysis.

## Conclusions

The use of stable isotopes analysis is a helpful technique to obtain quantitative estimates of the retention of different protein sources that can be distinguished based on their isotopic composition, such as FM and PBM. In the present work we report the potential total substitution of FM by PBM in trout diets, and shows that a high protein retention can be reached without an apparent effect on growth.
